# Supplementary material for: Pyrimethamine and a potent analog inhibit NRF2 by suppressing one-carbon metabolism
Source: J Biol Chem. 2025 Aug 30;301(10):110659. doi: 10.1016/j.jbc.2025.110659 (PMC12495326; doi:10.1016/j.jbc.2025.110659)
Supplement: Supplemental Figures [file mmc1.pdf]

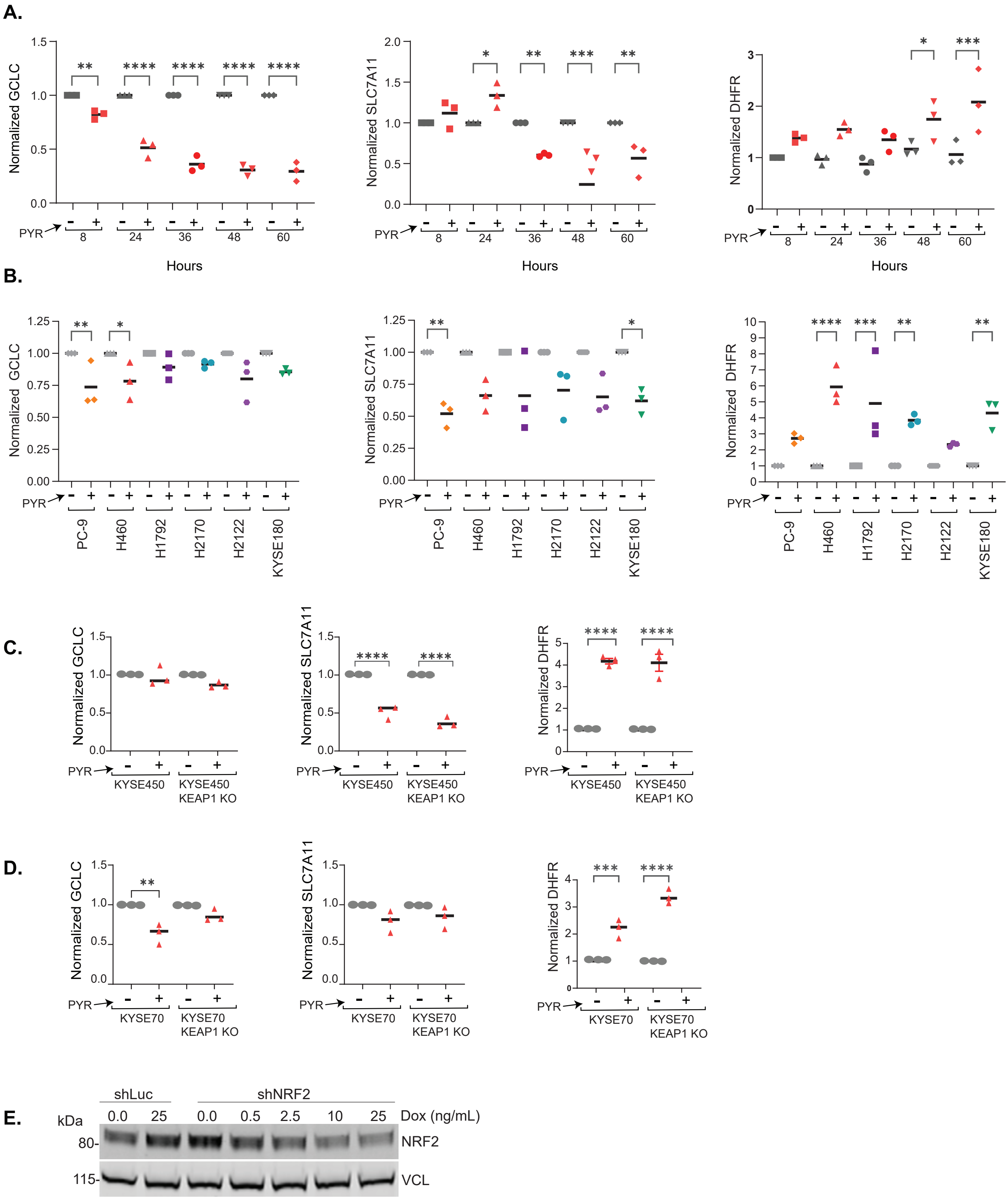

A.
Normalized GCLC
Normalized SLC7A11
Normalized DHFR
PYR
8
24
36
48
60
Hours
B.
Normalized GCLC
Normalized SLC7A11
Normalized DHFR
PYR
PC-9
H460
H1792
H2170
H2122
KYSE180
C.
Normalized GCLC
Normalized SLC7A11
Normalized DHFR
PYR
KYSE450
KYSE450 KEAP1 KO
D.
Normalized GCLC
Normalized SLC7A11
Normalized DHFR
PYR
KYSE70
KYSE70 KEAP1 KO
E.
shLuc
shNRF2
kDa
0.0
25
0.0
0.5
2.5
10
25
Dox (ng/mL)
80-
NRF2
115-
VCL

Supplemental Figure 1. Quantification of pyrimethamine-mediated suppression of NRF2 target proteins from Figure 1.

(A) Quantification of western blot analyses from KYSE70 cells treated with 10 μM pyrimethamine (PYR) for the indicated durations (blots shown in **Figure 1C**). Levels of GCLC, SLC7A11, and DHFR were normalized to vinculin (VCL) and plotted relative to vehicle control (n = 3 biological replicates). **(B)** Quantification of GCLC, SLC7A11, and DHFR protein levels across lung and esophageal cancer cell lines after 48 h PYR treatment (10 μM), corresponding to blots in **Figure 1D**. Values were normalized to VCL and shown relative to DMSO control (n = 3). **(C–D)** Quantification of target proteins GCLC, SLC7A11, and DHFR in KEAP1 knockout (KO) and parental KYSE450 (**C**) and KYSE70 (**D**) cells treated with PYR (10 μM, 48 h). These correspond to blots in **Figure 1E–F**. **(E)** Representative western blot of doxycycline-inducible NRF2 knockdown in KYSE70 cells (shNRF2) following 24 h Dox treatment, corresponding to protein quantification in **Figure 1H**. Data are presented as mean ± SD. Statistical analysis was performed using one-way ANOVA. *$P < 0.05$, **$P < 0.01$, ***$P < 0.001$, ****$P < 0.0001$.

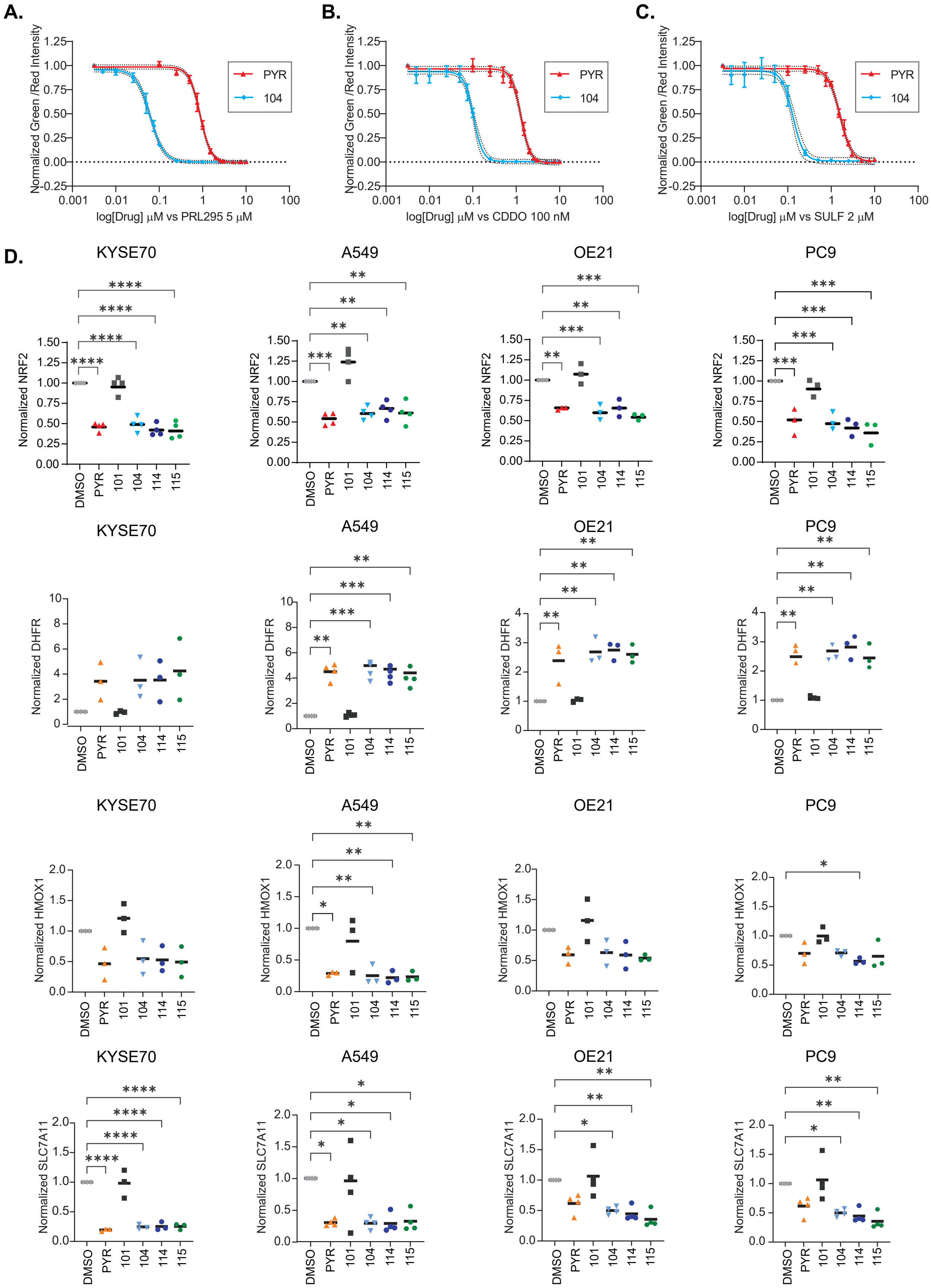

A.
B.
C.
Normalized Green /Red Intensity
PYR
104
log[Drug] µM vs PRL295 5 µM
log[Drug] µM vs CDDO 100 nM
log[Drug] µM vs SULF 2 µM
D.
KYSE70
A549
OE21
PC9
Normalized NRF2
Normalized DHFR
Normalized HMOX1
Normalized SLC7A11
DMSO
PYR
101
104
114
115

E.

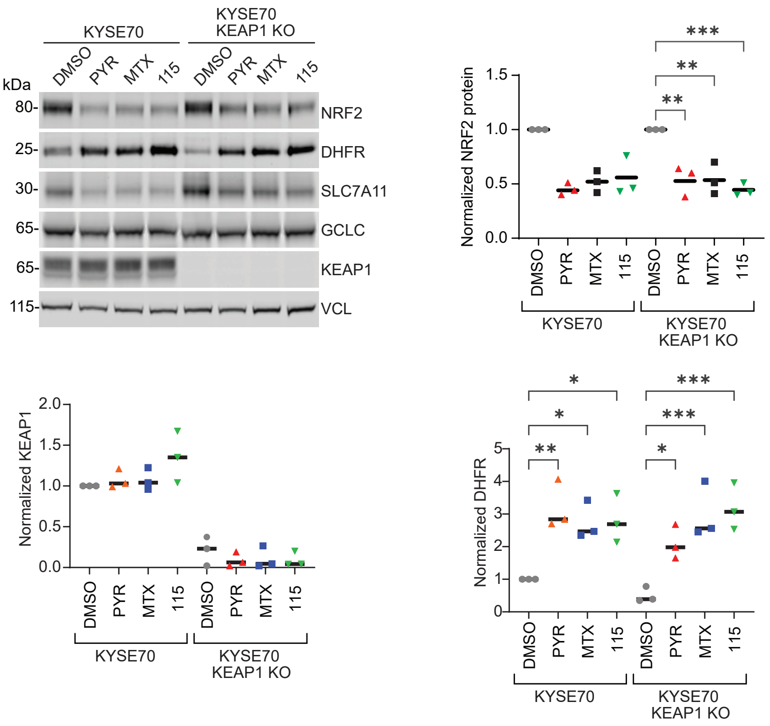
KYSE70
KYSE70 KEAP1 KO
DMSO PYR MTX 115 DMSO PYR MTX 115
kDa
80- NRF2
25- DHFR
30- SLC7A11
65- GCLC
65- KEAP1
115- VCL
Normalized NRF2 protein
Normalized KEAP1
Normalized DHFR
*
**
***

F.

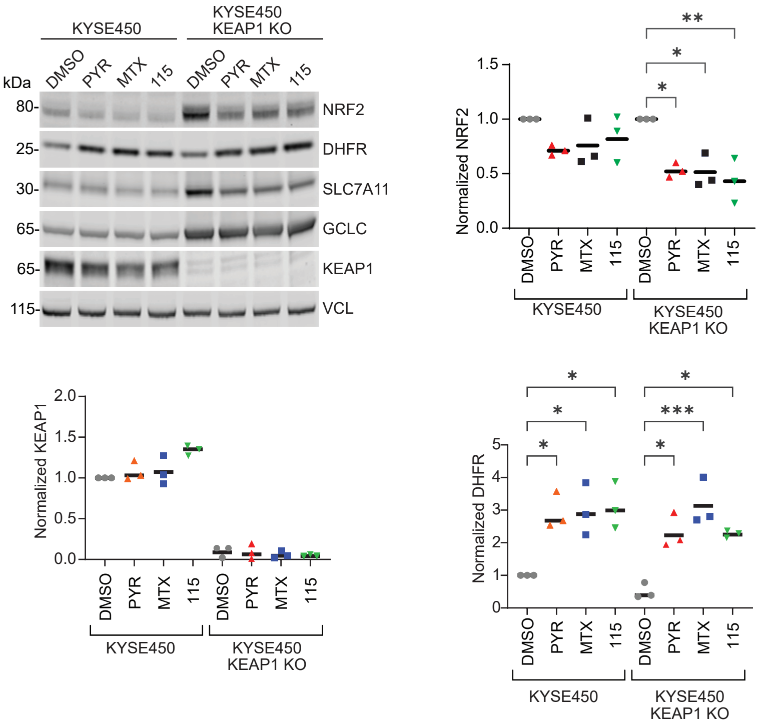
KYSE450
KYSE450 KEAP1 KO
DMSO PYR MTX 115 DMSO PYR MTX 115
kDa
80- NRF2
25- DHFR
30- SLC7A11
65- GCLC
65- KEAP1
115- VCL
Normalized NRF2
Normalized KEAP1
Normalized DHFR
*
**
***

Supplemental Figure 2. Quantitative analysis of NRF2 suppression by pyrimethamine analogs across cell lines and KEAP1 backgrounds. (A–C) Dose-response curves comparing the effects of WCDD104 and pyrimethamine (PYR) on suppression of NRF2-driven eYFP in H1299-NQO1-eYFP cells co-treated with 5 μM PRL-295 (A), 100 nM CDDOme (B), or 2 μM sulforaphane (SULF) (C). Data are normalized to mCherry fluorescence and presented as mean ± SD (n = 3). Corresponds to SAR results in **Figure 2A** and highlights increased potency of WCDD104 (IC_{50} = 0.098 μM). **(D)** Quantification of western blot analyses shown in **Figure 2E–H**, demonstrating effects of 48 h treatment with DMSO, PYR (10 μM), or 1 μM of WCDD101, WCDD104, WCDD114, or WCDD115 on endogenous NRF2 protein and its canonical targets (SLC7A11, HMOX1, GCLC, NQO1) across KYSE70, A549, OE21, and PC9 cells. Protein levels were normalized to vinculin (VCL) and plotted relative to DMSO control. **(E–F)** Representative western blots and quantification showing the effects of PYR and analogs on NRF2 protein expression of parental and KEAP1 knockout derivatives of KYSE70 (E) and KYSE450 (F) cells. NRF2, DHFR, and downstream targets (SLC7A11, GCLC) remain suppressed by active analogs irrespective of KEAP1 status. VCL served as loading control. Data are shown as mean ± SD from ≥3 independent biological replicates. Statistical analysis was performed using one-way ANOVA with multiple comparisons. *P < 0.05, **P < 0.01, ***P < 0.001, ****P < 0.0001.

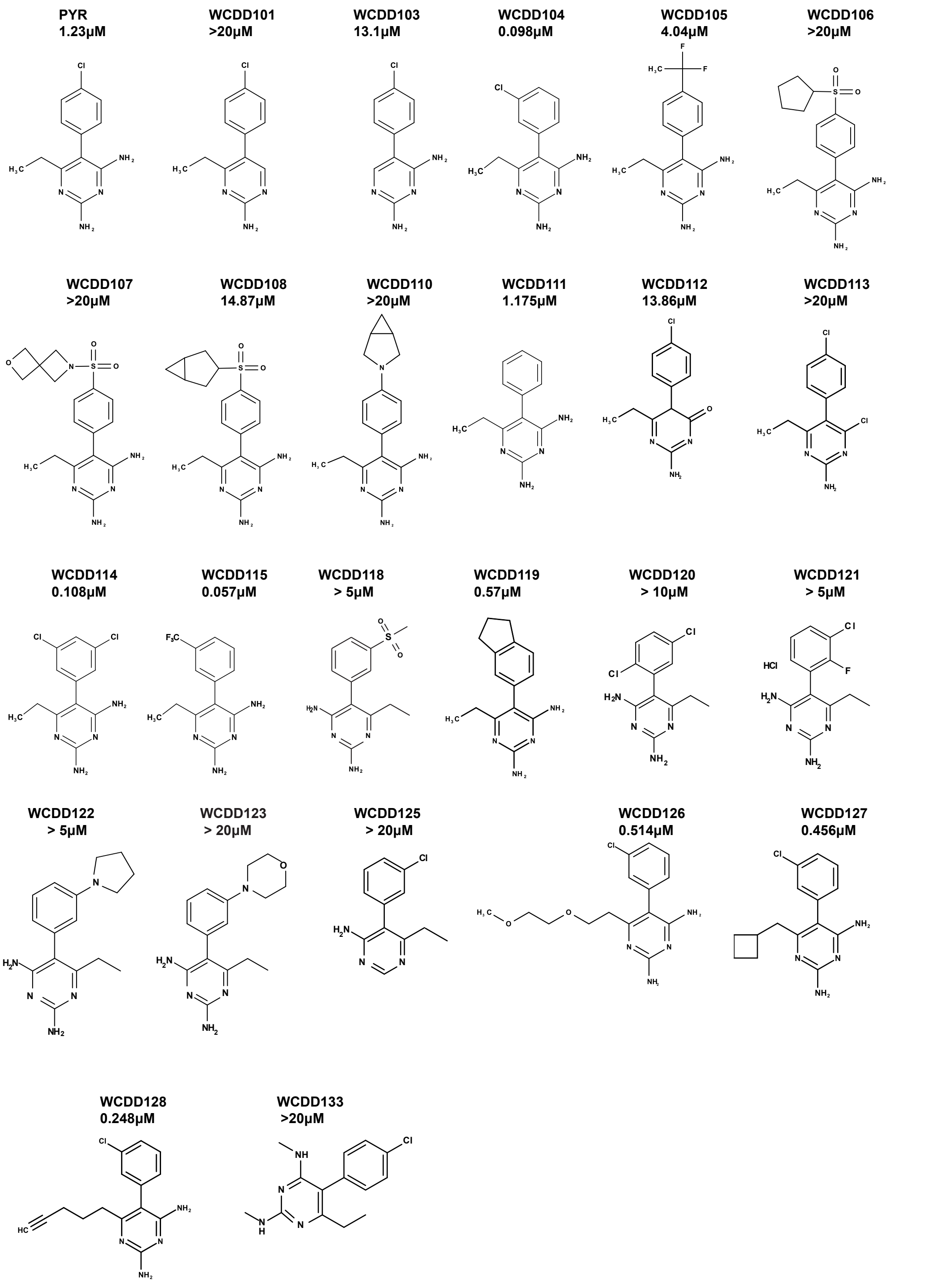

Supplemental Figure 3. Structures and NRF2 inhibition IC50 values of PYR analogues. IC50 values were defined by the H1299-NQO1-eYFP assay, taking the average IC50 values from SULF, PRL295 and CDDOme (see Table S2).

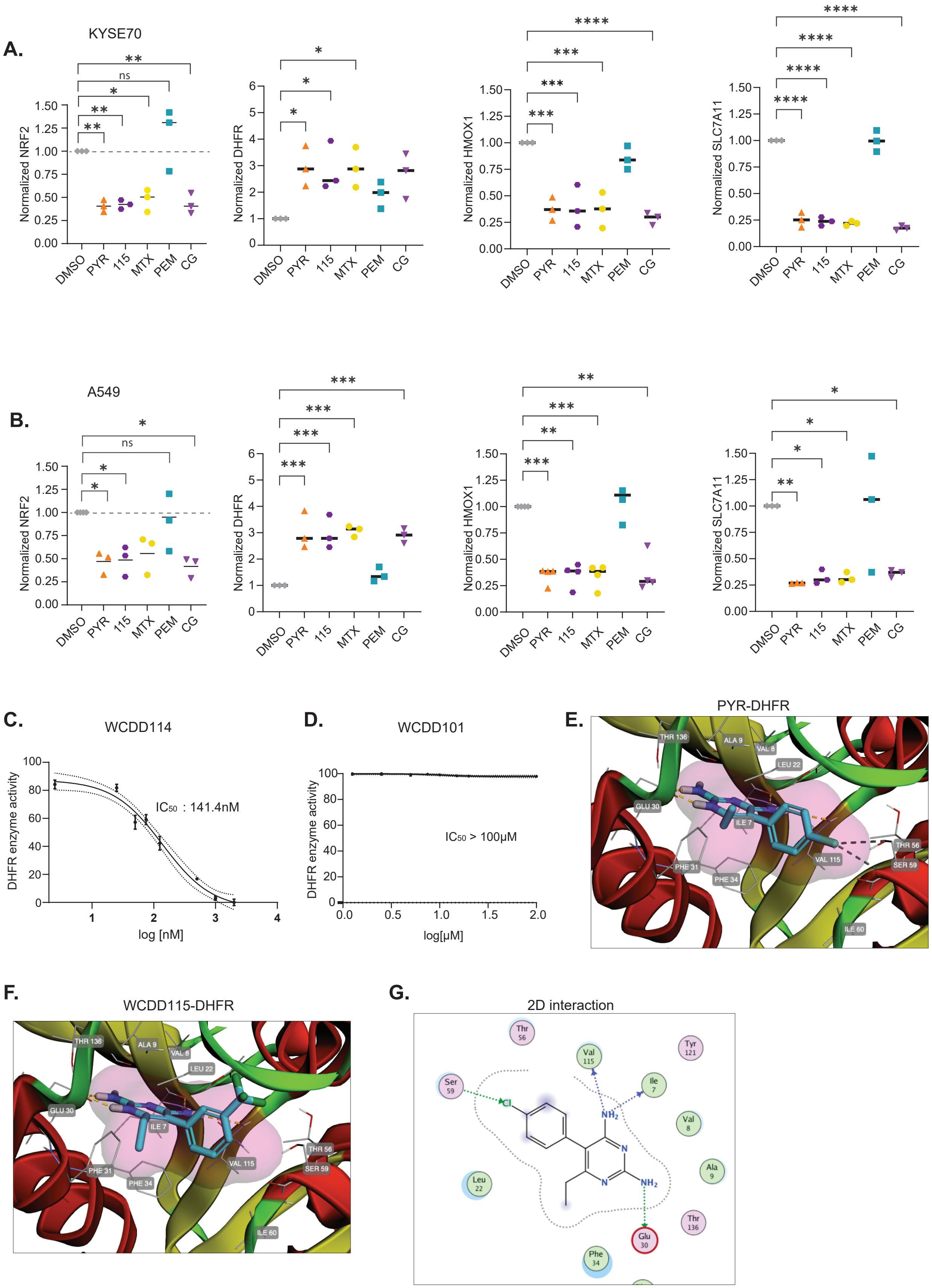

A.
KYSE70
Normalized NRF2
Normalized DHFR
Normalized HMOX1
Normalized SLC7A11
DMSO
PYR
115
MTX
PEM
CG
ns
B.
A549
C.
WCDD114
DHFR enzyme activity
IC50 : 141.4nM
log [nM]
D.
WCDD101
IC50 > 100μM
log[μM]
E.
PYR-DHFR
THR 136
ALA 9
VAL 8
LEU 22
GLU 30
ILE 7
THR 56
SER 59
VAL 115
PHE 31
PHE 34
ILE 60
F.
WCDD115-DHFR
G.
2D interaction
Thr 56
Val 115
Tyr 121
Ser 59
Ile 7
Val 8
Ala 9
Leu 22
Thr 136
Glu 30
Phe 34
Cl
NH2
N

Supplemental Figure 4. Quantitative validation of DHFR inhibition and structural modeling of WCDD analog interactions with human DHFR. (A–B) Quantification of western blot analyses shown in **Figure 3D–E**. Protein levels of NRF2, HMOX1, SLC7A11, and GCLC were quantified in KYSE70 (A) and A549 (B) cells after 48 h treatment with DMSO, PYR (10 μM), WCDD115 (1 μM), MTX (0.1 μM), PEM (100 nM), or cycloguanil (CG, 10 μM). Protein levels were normalized to VCL and plotted relative to DMSO. **(C–D)** Enzyme inhibition assays using recombinant human DHFR (3E–3 units). WCDD114 (C) inhibited DHFR activity with an IC_{50} of 141.4 nM, while WCDD101 (D), which lacks NRF2-suppressive activity, showed no detectable DHFR inhibition at concentrations up to 100 μM. Data represent biological triplicates. Statistical significance determined by one-way ANOVA (*$P < 0.05$, **$P < 0.01$). **(E,F)** Docking models of PYR and WCDD115 bound to the DHFR active site showing interactions with key residues. **(G)** 2D interaction map of PYR-DHFR illustrating hydrogen bonding interactions with Glu30, Ser59, Val115, and Ile7, consistent with its antifolate binding pose.

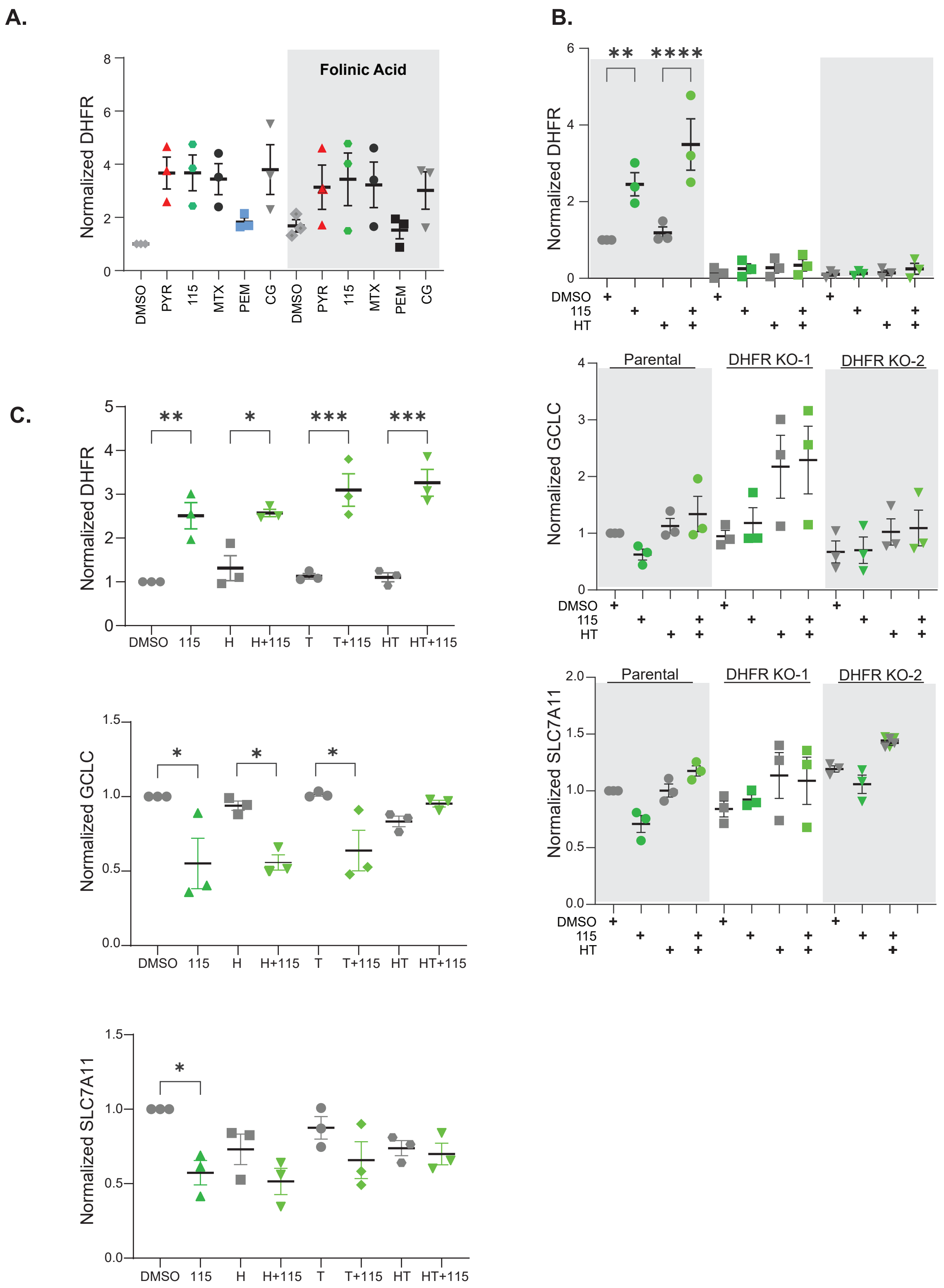
A.
Folinic Acid
Normalized DHFR
DMSO
PYR
115
MTX
PEM
CG
B.
Normalized DHFR
DMSO
115
HT
Parental
DHFR KO-1
DHFR KO-2
Normalized GCLC
Normalized SLC7A11
C.
Normalized DHFR
DMSO 115 H H+115 T T+115 HT HT+115
Normalized GCLC
Normalized SLC7A11

Supplemental Figure 5. Quantification of DHFR dependency and metabolic rescue experiments from Figure 4.

(A) Quantification of DHFR protein levels from western blots shown in **Figure 4A**, where KYSE70 cells were treated with DMSO, PYR (10 μM), WCDD115 (1 μM), MTX (0.1 μM), PEM (0.1 μM), or CG (10 μM) ± folinic acid (FA, 10 mg/mL). DHFR protein levels were normalized to VCL and expressed relative to DMSO control. **(B)** Quantification of DHFR, GCLC, and SLC7A11 protein levels from KYSE70 parental cells and two DHFR knockout clones (KO-1 and KO-2) shown in **Figure 4B**, following treatment with WCDD115 ± hypoxanthine and thymidine (HT) for 48 h. Data are normalized to total protein and plotted relative to DMSO. **(C)** Quantification of DHFR, GCLC, and SLC7A11 protein expression in KYSE70 cells treated with hypoxanthine (H), thymidine (T), or both (HT), with or without WCDD115 (1 μM), as shown in **Figure 4C**. Data represent mean ± SD from ≥3 biological replicates. Statistical analysis was performed using one-way ANOVA with multiple comparisons. *P < 0.05, **P < 0.01, ***P < 0.001, ****P < 0.0001.

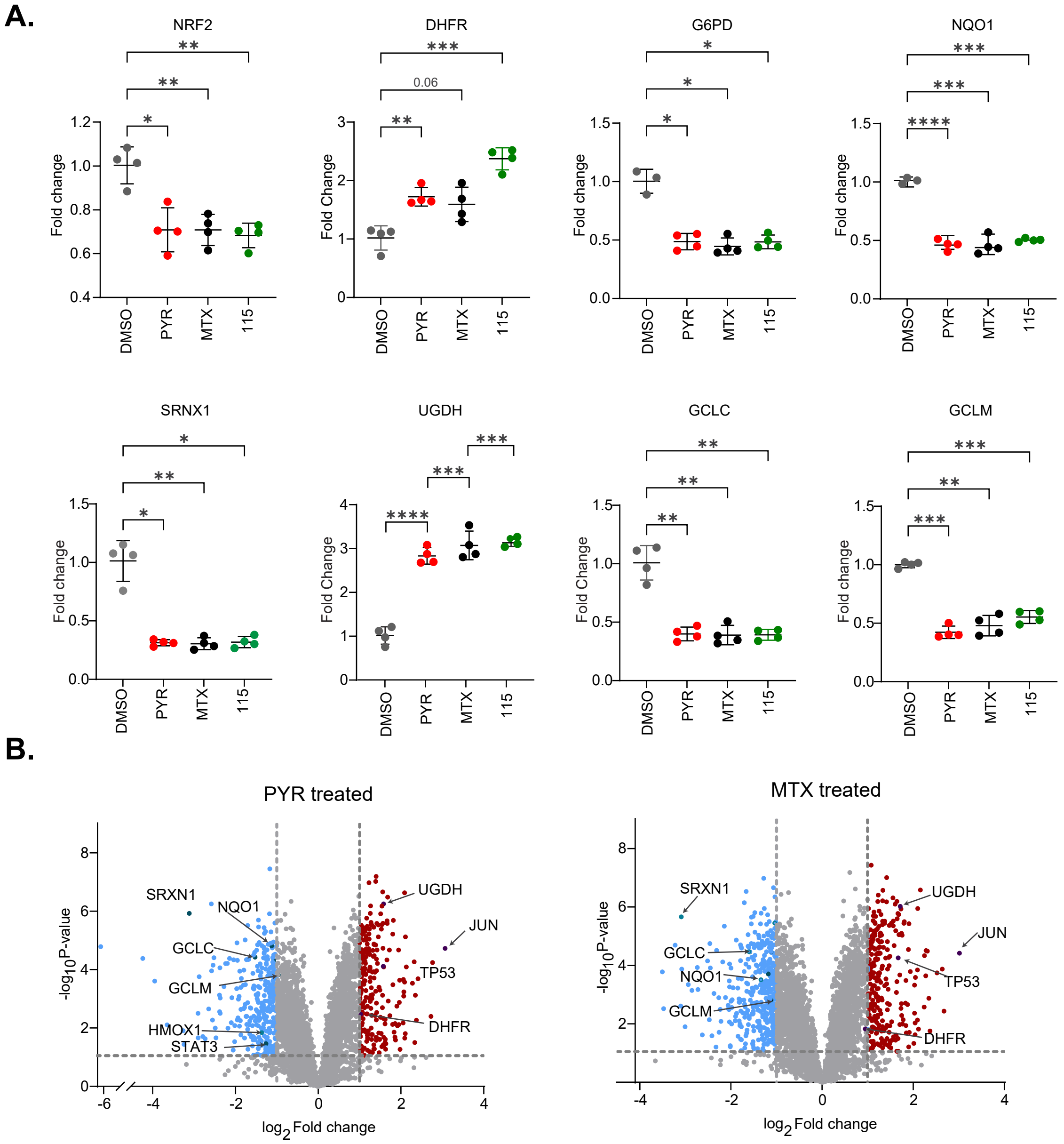

Supplemental Figure 6. **WCDD115 is an indirect inhibitor of NRF2**

(A) Quantification of selected proteins from the OIS-PRM proteomics experiment shown in **Figure 5A**, comparing KYSE70 cells treated for 48 h with DMSO, PYR (10 μM), WCDD115 (1 μM), or MTX (0.1 μM). Horizontal lines indicate mean values. Statistical comparisons were performed using one-way ANOVA.**(B)** Volcano plots showing differentially expressed proteins in KYSE70 cells treated with PYR or MTX for 48 h. Some NRF2 target genes are indicated. Data are presented as mean ± SD. *P < 0.05, **P < 0.01, ***P < 0.001, ****P < 0.0001.
