# Supplementary material for: Pyrimethamine and a potent analog inhibit NRF2 by suppressing one-carbon metabolism
Source: J Biol Chem. 2025 Aug 30;301(10):110659. doi: 10.1016/j.jbc.2025.110659 (PMC12495326; doi:10.1016/j.jbc.2025.110659)
Supplement: Supplemental methods [file mmc2.docx]

**Supplemental Methods**

5-(4-chlorophenyl)-4-ethylpyrimidin-2-amine (**WCDD101**)

To a solution of 4-chloro-5-(4-chlorophenyl)-6-ethyl-pyrimidin-2-amine **WCDD113** (20 mg, 74.59 umol, 1 *eq*) in MeOH (5 mL) was added TEA (15.09 mg, 149.18 umol, 20.76 uL, 2 *eq*) and Pd/C (10%, 0.01g) under N_2_ atmosphere. The suspension was degassed and purged with H_2_ for **3** times. The mixture was stirred under H_2_ (15 Psi.) at 25 °C for 1 hr. The mixture of the reaction was filtered through celite and concentrated to give a crude product, which was purified by prep-HPLC (HCl condition) (column: Welch Xtimate C18 100*25mm*3um;mobile phase: [water(0.05%HCl)-ACN];B%: 20%-50%,8min). Compound 5-(4-chlorophenyl)-4-ethyl-pyrimidin-2-amine **WCDD101** (5.1 mg, 21.56 umol, 28.91% yield, 98.8% purity) was obtained as a white solid. MS: *m/z* 234.0 (M+H)^+^. ^1^H NMR (400 MHz): MeOD δ 8.2 (s, 1H), 7.51-7.53 (d, J = 8 Hz, 1H), 7.37-7.39 (d, J = 8.8 Hz, 1H), 2.73-2.79 (m, 2H), 1.21 (t, 3H).

5-(4-chlorophenyl)pyrimidine-2,4-diamine (**WCDD103**)

A mixture of (4-chlorophenyl)boronic acid **WCDD103_4** (200 mg, 1.28 mmol, 1 *eq*) , 5-iodopyrimidine-2,4-diamine **WCDD103_3** (301.86 mg, 1.28 mmol, 1 *eq*) , K2CO3 (353.53 mg, 2.56 mmol, 2 *eq*) and Pd(dppf)Cl2 (140.38 mg, 191.85 umol, 0.15 *eq*) in dioxane (4 mL) and H2O (1 mL) was degassed and purged with N_2_ for 3 times, and then the mixture was stirred at 80 °C for 12 hr under N_2_ atmosphere. The reaction mixture was diluted with water (10 mL) and extracted with EtOAc (15 mL * 3). The combined organic layers were washed with brine (20 mL), dried over Na_2_SO_4_, filtered and concentrated under reduced pressure to give a residue, which was purified by prep-HPLC (column: Phenomenex luna C18 250*50mm*10 um;mobile phase: [water(0.04%HCl)-ACN];B%: 10%-40%,10min). Compound 5-(4-chlorophenyl)pyrimidine-2,4-diamine **WCDD103** (0.05 g, 226.60 umol, 17.72% yield, 100% purity) was obtained as a white solid. MS: *m/z* 221.0 (M+H)^+^. ^1^H NMR (400 MHz): MeOD δ 7.59 (s, 1H), 7.5 (d, J = 8.8 Hz, 2H), 7.70 (d, J = 8.4 Hz, 2H).

5-(3-chlorophenyl)-6-ethyl-pyrimidine-2,4-diamine (**WCDD104**)

A mixture of (3-chlorophenyl)boronic acid **WCDD104_1** (200 mg, 1.28 mmol, 1 *eq*) , 6-ethyl-5-iodo-pyrimidine-2,4-diamine **WCDD104_2** (337.74 mg, 1.28 mmol, 1 *eq*) , Pd(dppf)Cl2 (187.17 mg, 255.80 umol, 0.2 *eq*) and K2CO3 (353.53 mg, 2.56 mmol, 2 *eq*) in dioxane (4 mL)and H2O (1 mL) was degassed and purged with N_2_ for 3 times, and then the mixture was stirred at 80 °C for 12 hr under N_2_ atmosphere. The reaction mixture was diluted with water (10 mL) and extracted with EtOAc (15 mL * 3). The combined organic layers were washed with brine (20 mL), dried over Na_2_SO_4_, filtered and concentrated under reduced pressure to give a residue, which was purified by prep-HPLC (HCl condition) (column: Phenomenex luna C18 250*50mm*10 um;mobile phase: [water(0.04%HCl)-ACN];B%: 10%-40%,10min). Compound 5-(3-chlorophenyl)-6-ethyl-pyrimidine-2,4-diamine **WCDD104** (0.1 g, 402.07 umol, 31.44% yield, 100% purity) was obtained as a white solid. MS: *m/z* 249.0 (M+H)^+^. ^1^H NMR (400 MHz): MeOD δ 7.51-7.53 (d, 4.8 Hz, 2H), 7.36 (s, 1H), 7.24 (t, 1H), 2.31-2.37 (m, 2H), 1.11-1.15 (t, 3H).

5-[4-(1,1-difluoroethyl)phenyl]-6-ethyl-pyrimidine-2,4-diamine (**WCDD105**)

A mixture of 2-[4-(1,1-difluoroethyl)phenyl]-4,4,5,5-tetramethyl-1,3,2-dioxaborolane **WCDD105_1** (200 mg, 745.97 umol, 1 *eq*) , 6-ethyl-5-iodo-pyrimidine-2,4-diamine **WCDD104_2** (196.99 mg, 745.97 umol, 1 *eq*) , K2CO3 (206.20 mg, 1.49 mmol, 2 *eq*) and Pd(dppf)Cl2 (109.17 mg, 149.19 umol, 0.2 *eq*) in dioxane (4 mL) and H2O (1 mL) was degassed and purged with N_2_ for 3 times, and then the mixture was stirred at 80 °C for 12 hr under N_2_ atmosphere. The reaction mixture was diluted with water (10 mL) and extracted with EtOAc (15 mL * 3). The combined organic layers were washed with brine (20 mL), dried over Na_2_SO_4_, filtered and concentrated under reduced pressure to give a residue, which was purified by prep-HPLC (column: Phenomenex luna C18 250*50mm*10 um;mobile phase: [water(0.04%HCl)-ACN];B%: 10%-40%,10min). Compound 5-[4-(1,1-difluoroethyl)phenyl]-6-ethyl-pyrimidine-2,4-diamine **WCDD105** (0.05 g, 179.66 umol, 24.08% yield, 100% purity) was obtained as a white solid. MS: *m/z* 279.0 (M+H)^+^. ^1^H NMR (400 MHz): MeOD δ 7.70-7.72 (d, 8 Hz, 1H), 7.39-7.41 (d, 8 Hz, 1H), 2.31-2.37 (m, 2H), 1.92-2.01 (t, 3 H), 1.11-1.15 (t, 3H).

5-(4-cyclopentylsulfonylphenyl)-6-ethyl-pyrimidine-2,4-diamine (**WCDD106**)

To a solution of 4-bromobenzenethiol **WCDD106_1** (1 g, 5.29 mmol, 1 *eq*) in DMF (20 mL) was added K2CO3 (1.10 g, 7.93 mmol, 1.5 *eq*) and bromocyclopentane **WCDD106_2** (945.85 mg, 6.35 mmol, 680.47 uL, 1.2 *eq*). The mixture was stirred at 25 °C for 12 hr. The reaction mixture was concentrated under reduced pressure to remove DMF. The residue was diluted with water (100 mL) and extracted with EtOAc (30 mL * 3). The combined organic layers were washed with brine (30 mL), dried over Na_2_SO_4_, filtered and concentrated under reduced pressure to give Compound 1-bromo-4-cyclopentylsulfanyl-benzene **WCDD106_3** (1 g, 3.89 mmol, 73.52% yield) as a colourless oil. ^1^H NMR (400 MHz): CDCl_3_ δ 7.34-7.41 (m, 8 Hz, 2H), 7.19-7.21 (d, J = 8.3 Hz, 2H), 3.55 (td, 1H), 2.03-2.09 (m, 2H), 1.88-1.90 (td, 1H), 1.58-1.77 (s, 1H), 1.23-1.27 (s, 4H).

To a solution of 1-bromo-4-cyclopentylsulfanyl-benzene **WCDD106_3** (0.2 g, 777.64 umol, 1 *eq*) in DCM (5 mL) was added m-CPBA (258.07 mg, 1.17 mmol, 78% purity, 1.5 *eq*). The mixture was stirred at 25 °C for 12 hr. The reaction mixture was diluted with water (10 mL) and extracted with DCM (10 mL * 3). The combined organic layers were washed with brine (20 mL), dried over Na_2_SO_4_, filtered and concentrated under reduced pressure to give a residue, which was purified by prep-TLC (SiO2, PE : EtOAc= 4**:1**). Compound 1-bromo-4-cyclopentylsulfonyl-benzene **WCDD106_4** (40 mg, 124.49 umol, 16.01% yield, 90% purity) was obtained as a colourless oil. ^1^H NMR (400 MHz): CDCl_3_ δ 7.65-7.7 (d, 2H), 7.6-7.7 (d, 2H), 3.35 (m, 1H), 1.95-2.05 (dt, 2H), 1.75-1.80 (dt, 2H), 1.65-1.70 (dt, 2H), 1.14-1.55 (dt, 2H)

A mixture of 1-bromo-4-cyclopentylsulfonyl-benzene **WCDD106_4** (0.5 g, 1.73 mmol, 1 *eq*) , BPD (1.32 g, 5.19 mmol, 3 *eq*) , AcOK (339.37 mg, 3.46 mmol, 2 *eq*) and Pd(dppf)Cl2 (126.51 mg, 172.90 umol, 0.1 *eq*) in dioxane (4 mL) was degassed and purged with N_2_ for 3 times, and then the mixture was stirred at 80 °C for 12 hr under N_2_ atmosphere. The reaction mixture was diluted with water (10 mL) and extracted with EtOAc (10 mL * 3). The combined organic layers were washed with brine (20 mL), dried over Na_2_SO_4_, filtered and concentrated under reduced pressure to give a residue, which was purified by prep-TLC (SiO2, PE: EtOAc = 1:1). Compound 2-(4-cyclopentylsulfonylphenyl)-4,4,5,5-tetramethyl-1,3,2-dioxaborolane **WCDD106_5** (0.1 g, 237.92 umol, 13.76% yield, 80% purity) was obtained as a white solid. ^1^H NMR (400 MHz): CDCl_3_ δ 7.96-7.98 (d, 2H), 7.87-7.89 (d, 2H), 3.35 (m, 1H), 2.03-2.08 (m, 2 H), 1.61-1.77 (m, 4 H), 1.58-1.61 (m, 2 H), 1.36 (s, 12 H)

A mixture of 2-(4-cyclopentylsulfonylphenyl)-4,4,5,5-tetramethyl-1,3,2-dioxaborolane **WCDD106_5** (140 mg, 416.35 umol, 1 *eq*) , 6-ethyl-5-iodo-pyrimidine-2,4-diamine **WCDD104_2** (131.93 mg, 499.62 umol, 1.2 *eq*) , Pd(dppf)Cl2 (30.46 mg, 41.64 umol, 0.1 *eq*) and K2CO3 (115.09 mg, 832.71 umol, 2 *eq*) in dioxane (4 mL) and H2O (1 mL) was degassed and purged with N_2_ for 3 times, and then the mixture was stirred at 80 °C for 12 hr under N_2_ atmosphere. The reaction mixture was diluted with water (10 mL) and extracted with EtOAc (10 mL * 3). The combined organic layers were washed with brine (20 mL), dried over Na_2_SO_4_, filtered and concentrated under reduced pressure to give a residue, which was purified by prep-HPLC (column: Phenomenex luna C18 80*40mm*3 um;mobile phase: [water(0.04%HCl)-ACN];B%: 23%-35%,4min). Compound 5-(4-cyclopentylsulfonylphenyl)-6-ethyl-pyrimidine-2,4-diamine **WCDD106** (26 mg, 73.33 umol, 17.61% yield, 97.71% purity) was obtained as a white solid. MS: *m/z* 347.0 (M+H)^+^. ^1^H NMR (400 MHz): MeOD δ 8.05-8.07 (d, 2H), 7.58-7.60 (d, 2H), 3.75 (m, 1H), 2.34 (td, 2H), 2.03-2.08 (m, 2 H), 1.61-1.77 (m, 4 H), 1.58-1.61 (m, 2 H), 1.11-1.15 (t, 3H)

6-ethyl-5-[4-(2-oxa-6-azaspiro[3.3]heptan-6-ylsulfonyl)phenyl]pyrimidine-2,4-diamine (**WCDD107**)

To a solution of 4-bromobenzenesulfonyl chloride **WCDD107_1** (414.58 mg, 1.62 mmol, 1.1 *eq*) in DCM (5 mL) was added TEA (597.02 mg, 5.90 mmol, 821.21 uL, 4 *eq*) and 2-oxa-6-azaspiro[3.3]heptane **WCDD107_2** (0.2 g, 1.48 mmol, 1 *eq*, HCl). The mixture was stirred at 25 °C for 12 hr. The mixture of the reaction was concentrated to give the residue, which was purified by column chromatography (SiO2, Petroleum ether/Ethyl acetate=50/1 to 10/1). Compound 6-(4-bromophenyl)sulfonyl-2-oxa-6-azaspiro[3.3]heptane **WCDD107_3** (0.3 g, 848.56 umol, 57.53% yield, 90% purity) was obtained as a white solid. MS: *m/z* 317.7 (M+H)^+^. ^1^H NMR (400 MHz): CDCl_3_ δ 7.65-7.75 (td, 4H), 4.65-4.70 (s, 4H), 3.90-4.00 (s, 4H).

A mixture of 6-(4-bromophenyl)sulfonyl-2-oxa-6-azaspiro[3.3]heptane **WCDD107_3** (0.23 g, 722.85 umol, 1 *eq*) , BPD (550.67 mg, 2.17 mmol, 3 *eq*) , Pd(dppf)Cl2 (52.89 mg, 72.28 umol, 0.1 *eq*) and AcOK (141.88 mg, 1.45 mmol, 2 *eq*) in dioxane (4 mL) was degassed and purged with N_2_ for 3 times, and then the mixture was stirred at 80 °C for 12 hr under N_2_ atmosphere. The reaction mixture was diluted with water (10 mL) and extracted with EtOAc (10 mL * 3). The combined organic layers were washed with brine (20 mL), dried over Na_2_SO_4_, filtered and concentrated under reduced pressure to give a residue, which was purified by prep-TLC (SiO2, PE: EtOAc = 2:1). Compound 6-[4-(4,4,5,5-tetramethyl-1,3,2-dioxaborolan-2-yl)phenyl]sulfonyl-2-oxa-6-azaspiro[3.3]heptane **WCDD107_4** (0.1 g, 273.78 umol, 37.88% yield) was obtained as a white solid. ^1^H NMR (400 MHz): CDCl_3_ δ 7.99-8.01 (d, 2H), 7.79-7.81 (d, 2H), 4.58 (s, 4H), 3.92 (s, 4H), 1.37 (s, 12H).

A mixture of 6-[4-(4,4,5,5-tetramethyl-1,3,2-dioxaborolan-2-yl)phenyl]sulfonyl-2-oxa-6-azaspiro[3.3]heptane **WCDD107_4** (70 mg, 191.65 umol, 1 *eq*) , 6-ethyl-5-iodo-pyrimidine-2,4-diamine **WCDD104_2** (55.67 mg, 210.81 umol, 1.1 *eq*) , Pd(dppf)Cl2 (14.02 mg, 19.16 umol, 0.1 *eq*) and K2CO3 (52.97 mg, 383.30 umol, 2 *eq*) in dioxane (4 mL) and H2O (1 mL) was degassed and purged with N_2_ for 3 times, and then the mixture was stirred at 80 °C for 12 hr under N_2_ atmosphere. The reaction mixture was diluted with water (10 mL) and extracted with EtOAc (10 mL * 3). The combined organic layers were washed with brine (20 mL), dried over Na_2_SO_4_, filtered and concentrated under reduced pressure to give a residue, which was purified by prep-HPLC. (column: Welch Xtimate C18 100*25mm*3um;mobile phase: [water(0.05%HCl)-ACN];B%: 10%-30%,8min). Compound 6-ethyl-5-[4-(2-oxa-6-azaspiro[3.3]heptan-6-ylsulfonyl)phenyl]pyrimidine-2,4-diamine **WCDD107** (17.5 mg, 44.28 umol, 23.11% yield, 95% purity) was obtained as a white solid. MS: *m/z* 376.0 (M+H)^+^. ^1^H NMR (400 MHz): MeOD δ 7.99-8.01 (d, 2H), 7.59-7.61 (d, 2H), 4.58 (s, 4H), 4.02 (s, 4H), 2.34 (m, 2H), 1.12 (t, 3H).

5-[4-(3-azabicyclo[3.1.0]hexan-3-ylsulfonyl)phenyl]-6-ethyl-pyrimidine-2,4-diamine (**WCDD108**)

To a solution of 4-bromobenzenesulfonyl chloride **WCDD107_1** (427.31 mg, 1.67 mmol, 1 *eq*) in DCM (4 mL) was added TEA (676.89 mg, 6.69 mmol, 931.08 uL, 4 *eq*) and 3-azabicyclo[3.1.0]hexane **WCDD108_1** (200 mg, 1.67 mmol, 1 *eq*, HCl). The mixture was stirred at 25 °C for 12 hr. The mixture of the reaction was concentrated to give a crude product, which was purified by column chromatography (SiO2, Petroleum ether/Ethyl acetate=50/1 to 20/1). Compound 3-(4-bromophenyl)sulfonyl-3-azabicyclo[3.1.0]hexane **WCDD108_2** (0.3 g, 794.21 umol, 47.49% yield, 80% purity) was obtained as a white solid. ^1^H NMR (400 MHz): CDCl_3_ δ 7.65-7.75 (d, 2H), 3.50-3.55 (d, 2H), 3.05-3.10 (d, 2H), 1.49 (m, 2H), 0.55-0.65 (m, 1H), 0.30-0.40 (m, 1H).

A mixture of 3-(4-bromophenyl)sulfonyl-3-azabicyclo[3.1.0]hexane **WCDD108_2** (50 mg, 165.46 umol, 1 *eq*) , BPD (126.05 mg, 496.38 umol, 3 *eq*) , AcOK (32.48 mg, 330.92 umol, 2 *eq*) and Pd(dppf)Cl2 (12.11 mg, 16.55 umol, 0.1 *eq*) in dioxane (4 mL) was degassed and purged with N_2_ for 3 times, and then the mixture was stirred at 80 °C for 12 hr under N_2_ atmosphere. The reaction mixture was diluted with water (10 mL) and extracted with EtOAc (10 mL * 3). The combined organic layers were washed with brine (20 mL), dried over Na_2_SO_4_, filtered and concentrated under reduced pressure to give a residue, which was purified by prep-TLC (SiO2, PE: EtOAc= 2:1). Compound 3-[4-(4,4,5,5-tetramethyl-1,3,2-dioxaborolan-2-yl)phenyl]sulfonyl-3-azabicyclo[3.1.0]hexane **WCDD108_3** (10 mg, 28.63 umol, 17.30% yield) was obtained as a white solid. ^1^H NMR (400 MHz): CDCl_3_ δ 7.95-7.97 (d, 2H), 7.77-7.79 (d, 2H), 3.51-3.54 (d, 2H), 3.05-3.07 (d, 2H), 1.36 (s, 12H), 1.27 (s, 2H), 0.55 (m, 1H), 0.34 (m, 1H).

A mixture of 3-[4-(4,4,5,5-tetramethyl-1,3,2-dioxaborolan-2-yl)phenyl]sulfonyl-3-azabicyclo[3.1.0]hexane **WCDD108_3** (95 mg, 272.01 umol, 1 *eq*) , 6-ethyl-5-iodo-pyrimidine-2,4-diamine **WCDD104_2** (86.19 mg, 326.41 umol, 1.2 *eq*) , Pd(dppf)Cl2 (19.90 mg, 27.20 umol, 0.1 *eq*) and K2CO3 (75.19 mg, 544.02 umol, 2 *eq*) in dioxane (4 mL) and H2O (1 mL) was degassed and purged with N_2_ for 3 times, and then the mixture was stirred at 80 °C for 12 hr under N_2_ atmosphere. The reaction mixture was diluted with water (10 mL) and extracted with EtOAc (10 mL * 3). The combined organic layers were washed with brine (20 mL), dried over Na_2_SO_4_, filtered and concentrated under reduced pressure to give a residue, which was purified by prep-HPLC. (column: Phenomenex luna C18 80*40mm*3 um;mobile phase: [water(0.04%HCl)-ACN];B%: 22%-37%,4.5min). Compound 5-[4-(3-azabicyclo[3.1.0]hexan-3-ylsulfonyl)phenyl]-6-ethyl-pyrimidine-2,4-diamine **WCDD108** (40 mg, 108.93 umol, 40.05% yield, 97.89% purity) was obtained as a white solid. ^1^H NMR (400 MHz): MeOD δ 7.93-7.95 (d, 2H), 7.56-7.54 (d, 2H), 3.50-3.53 (d, 2H), 3.15-3.17 (d, 2H), 2.32 (m, 2H), 1.51 (td, 2H), 1.13 (t, 3H), 0.61 (m, 1H), 0.29 (m, 1H).

5-[4-(3-azabicyclo[3.1.0]hexan-3-yl)phenyl]-6-ethyl-pyrimidine-2,4-diamine (**WCDD110**)

A mixture of 1-bromo-4-iodo-benzene **WCDD110_1** (1.02 g, 3.61 mmol, 1 *eq*) , 3-azabicyclo[3.1.0]hexane **WCDD108_1** (300 mg, 3.61 mmol, 1 *eq*) , Pd(dba)2 (62.25 mg, 108.26 umol, 0.03 *eq*) , dicyclohexyl-[2-(2,4,6-triisopropylphenyl)phenyl]phosphane (120.42 mg, 252.61 umol, 0.07 *eq*) and Cs2CO3 (2.94 g, 9.02 mmol, 2.5 *eq*) in Tol. (6 mL) was degassed and purged with N_2_ for 3 times, and then the mixture was stirred at 100 °C for 12 hr under N_2_ atmosphere. The reaction mixture was diluted with water (10 mL) and extracted with EtOAc (10 mL * 3). The combined organic layers were washed with brine (20 mL), dried over Na_2_SO_4_, filtered and concentrated under reduced pressure to give a residue, which was purified by prep-TLC (SiO2, PE: EtOAc = 5:1). Compound 3-(4-bromophenyl)-3-azabicyclo[3.1.0]hexane **WCDD110_2** (0.25 g, 944.89 umol, 26.18% yield, 90% purity) was obtained as a light yellow solid.

A mixture of 3-(4-bromophenyl)-3-azabicyclo[3.1.0]hexane **WCDD110_2** (260 mg, 1.09 mmol, 1 *eq*) , BPD (831.80 mg, 3.28 mmol, 3 *eq*) , Pd(dppf)Cl2 (79.89 mg, 109.19 umol, 0.1 *eq*) and KOAc (267.90 mg, 2.73 mmol, 2.5 *eq*) in dioxane (4 mL) was degassed and purged with N_2_ for 3 times, and then the mixture was stirred at 80 °C for 12 hr under N_2_ atmosphere. The reaction mixture was diluted with water (10 mL) and extracted with EtOAc (10 mL * 3). The combined organic layers were washed with brine (20 mL), dried over Na_2_SO_4_, filtered and concentrated under reduced pressure to give a residue, which was purified by prep-TLC (SiO2, PE: EtOAc = 2:1). Compound 3-[4-(4,4,5,5-tetramethyl-1,3,2-dioxaborolan-2-yl)phenyl]-3-azabicyclo[3.1.0]hexane **WCDD110_3** (0.2 g, 631.16 umol, 57.81% yield, 90% purity) was obtained as a white solid. ^1^H NMR (400 MHz): CDCl­_3_ δ 7.64-7.66 (d, 2H), 6.51-6.53 (d, 2H), 3.53-3.55 (d, 2H), 3.28-3.30 (d, 2H), 1.63-1.65 (m, 2H), 1.28 (s, 12H), 0.73 (m, 1H), 0.31 (m, 1H).

A mixture of 3-[4-(4,4,5,5-tetramethyl-1,3,2-dioxaborolan-2-yl)phenyl]-3-azabicyclo[3.1.0]hexane **WCDD110_3** (0.2 g, 701.29 umol, 1 *eq*) , 6-ethyl-5-iodo-pyrimidine-2,4-diamine **WCDD104_2** (222.22 mg, 841.55 umol, 1.2 *eq*) , Pd(dppf)Cl2 (51.31 mg, 70.13 umol, 0.1 *eq*) and K2CO3 (193.85 mg, 1.40 mmol, 2 *eq*) in dioxane (4 mL) and H2O (1 mL) was degassed and purged with N_2_ for 3 times, and then the mixture was stirred at 80 °C for 12 hr under N_2_ atmosphere. The reaction mixture was diluted with water (10 mL) and extracted with EtOAc (10 mL * 3). The combined organic layers were washed with brine (20 mL), dried over Na_2_SO_4_, filtered and concentrated under reduced pressure to give a residue, which was purified by prep-HPLC.(column: Xtimate C18 100*30mm*3um;mobile phase: [water(0.04%HCl)-ACN];B%: 24%-44%,8min). Compound 5-[4-(3-azabicyclo[3.1.0]hexan-3-yl)phenyl]-6-ethyl-pyrimidine-2,4-diamine **WCDD110** (25 mg, 84.64 umol, 12.07% yield, 100% purity) was obtained as a white solid. MS: *m/z* 296.1 (M+H)^+^. ^1^H NMR (400 MHz): MeOD δ 7.08-7.10 (d, 2H), 6.80-6.83 (d, 2H), 3.59-3.62 (d, 2H), 3.34 (m, 2H), 2.35 (q, 2H), 1.75 (m, 2H), 1.12 (t, 3H), 0.79 (m, 1H), 0.38 (m, 1H).

6-ethyl-5-phenyl-pyrimidine-2,4-diamine (**WCDD111**)

A mixture of phenylboronic acid **WCDD111_1** (200 mg, 1.64 mmol, 1 *eq*) , 6-ethyl-5-iodo-pyrimidine-2,4-diamine **WCDD104_2** (433.15 mg, 1.64 mmol, 1 *eq*) , K2CO3 (453.40 mg, 3.28 mmol, 2 *eq*) and Pd(dppf)Cl2 (240.04 mg, 328.06 umol, 0.2 *eq*) and in dioxane (4 mL) and H2O (1 mL) was degassed and purged with N_2_ for 3 times, and then the mixture was stirred at 80 °C for 12 hr under N_2_ atmosphere. The reaction mixture was diluted with water (10 mL) and extracted with EtOAc (15 mL * 3). The combined organic layers were washed with brine (20 mL), dried over Na_2_SO_4_, filtered and concentrated under reduced pressure to give a residue, which was purified by prep-HPLC.(column: Phenomenex luna C18 250*50mm*10 um;mobile phase: [water(0.04%HCl)-ACN];B%: 5%-35%,10min). Compound 6-ethyl-5-phenyl-pyrimidine-2,4-diamine **WCDD111** (0.05 g, 233.36 umol, 14.23% yield, 100% purity) was obtained as a white solid. MS: *m/z* 215.0 (M+H)^+^. ^1^H NMR (400 MHz): MeOD δ 7.54 (m, 3H), 7.29 (d, 2H), 2.34 (q, 2H), 1.12 (t, 3H).

2-amino-5-(4-chlorophenyl)-6-ethyl-5H-pyrimidin-4-one (**WCDD112**)

To a solution of 5-(4-chlorophenyl)-6-ethyl-pyrimidine-2,4-diamine **WCDD101_1** (5 g, 20.10 mmol, 1 *eq*) was added HCl (12 M, 83.77 mL, 50 *eq*). The mixture was stirred at 100 °C for 48 hr. The mixture of the reaction was concentrated to give a crude product, which was purified by prep-HPLC twice. (column: Phenomenex luna c18 250mm*100mm*10um;mobile phase: [water(0.05%HCl)-ACN];B%: 1%-30%,25min) and (column: Agela DuraShell C18 250*70mm*10um;mobile phase: [water(10mM NH4HCO3)-ACN];B%: 20%-40%,20min). Compound 2-amino-5-(4-chlorophenyl)-6-ethyl-5H-pyrimidin-4-one **WCDD112** (1.5 g, 5.41 mmol, 26.89% yield, 90% purity) was obtained as a white solid. MS: *m/z* 249.9 (M+H)^+^. ^1^H NMR (400 MHz): DMSO-d6 δ 12.20 (s, 2H), 8.52 (s, 1H), 8.08 (s, 1H), 7.57 (d, 2H), 7.34 (d, 2H), 2.15 (q, 2H), 0.99 (t, 3H).

4-chloro-5-(4-chlorophenyl)-6-ethylpyrimidin-2-amine (**WCDD113**)

To a solution of 4-chloro-6-ethyl-pyrimidin-2-amine **WCDD113_1** (0.3 g, 1.90 mmol, 1 *eq*) in AcOH (10 mL) was added NIS (471.09 mg, 2.09 mmol, 1.1 *eq*) at 0°C. The mixture was stirred at 25°C for 3 hr. The reaction mixture was diluted with water (20 mL) and 10% Na_2_S_2_SO_3_ aq was added until the color of the mixture disappeared. The solid was filtered. Compound 4-chloro-6-ethyl-5-iodo-pyrimidin-2-amine **WCDD113_2** (0.25 g, 837.75 umol, 44.01% yield, 95% purity) was obtained as a white solid. MS: *m/z* 283.8 (M+H)^+^.

A mixture of 4-chloro-6-ethyl-5-iodo-pyrimidin-2-amine **WCDD113_2** (220 mg, 776.02 umol, 1 *eq*) , (4-chlorophenyl)boronic acid **WCDD103_4** (145.62 mg, 931.23 umol, 1.2 *eq*) , Pd(dppf)Cl_2_.CH_2_Cl_2_ (63.37 mg, 77.60 umol, 0.1 *eq*) and K3PO4 (494.18 mg, 2.33 mmol, 3 *eq*) in dioxane (4 mL) was degassed and purged with N_2_ for 3 times, and then the mixture was stirred at 80 °C for 12 hr under N_2_ atmosphere. The reaction mixture was diluted with water (10 mL) and extracted with EtOAc (10 mL * 3). The combined organic layers were washed with brine (20 mL), dried over Na_2_SO_4_, filtered and concentrated under reduced pressure to give a residue. The residue was purified by prep-HPLC. (column: Phenomenex luna C18 80*40mm*3 um;mobile phase: [water(0.04%HCl)-ACN];B%: 40%-65%,7min). Compound 4-chloro-5-(4-chlorophenyl)-6-ethyl-pyrimidin-2-amine **WCDD113** (120 mg, 425.15 umol, 54.79% yield, 95% purity) was obtained as a white solid. MS: *m/z* 267.8 (M+H)^+^. ^1^H NMR (400 MHz): CDCl_3_ δ 7.45-7.50 (d, 2H), 7.10-7.15 (d, 2H), 2.51 (q, 2H), 1.22 (t, 3H)

5-(3,5-dichlorophenyl)-6-ethyl-pyrimidine-2,4-diamine (**WCDD114**)

To a mixture of 2-(3,5-dichlorophenyl)-4,4,5,5-tetramethyl-1,3,2-dioxaborolane **WCDD114_1** (200 mg, 732.70 umol, 1 *eq*) and 6-ethyl-5-iodo-pyrimidine-2,4-diamine **WCDD114_2** (193.48 mg, 732.70 umol, 1 *eq*) in dioxane (10 mL) and H2O (2.5 mL) was added Pd(dppf)Cl2 (53.61 mg, 73.27 umol, 0.1 *eq*) and K2CO3 (202.53 mg, 1.47 mmol, 2 *eq*) in one portion at 25°C under N_2_.The mixture was heated to 80 °C and stirred for 10 hr. The reaction mixture was poured into H_2_O (5 mL). The mixture was extracted with ethyl acetate (10 mL*3). The organic phase was washed with brine (10 mL), dried over anhydrous Na2SO_4_, concentrated in vacuum to give a residue, which was purified by column chromatography to give 5-(3,5-dichlorophenyl)-6-ethyl-pyrimidine-2,4-diamine **WCDD114** (33.6 mg, 117.29 umol, 16.01% yield, 98.84% purity) as a white solid. MS: *m/z* 283.0 (M+H)^+^. ^1^H NMR (400 MHz): DMSO δ 7.489 (s, 1H), 7.222-7.224 (d, J = 0.8 Hz, 2H), 2.218-2.238 (q, J = 8 Hz, 2H), 1.040-1.078 (t, J = 15.2 Hz, 3H). ^13^C NMR (400 MHz): MeOD_4_ δ 167.13, 162.54, 162.10, 138.77, 135.34, 129.23, 127.57, 105.35, 27.27, 12.24.

6-ethyl-5-[3-(trifluoromethyl)phenyl]pyrimidine-2,4-diamine (**WCDD115**)

[3-(trifluoromethyl)phenyl]boronic acid **WCDD115_1** (200 mg, 1.05 mmol, 1 *eq*) , 6-ethyl-5-iodo-pyrimidine-2,4-diamine **WCDD114_2** (278.07 mg, 1.05 mmol, 1 *eq*) ,K3PO4 (447.05 mg, 2.11 mmol, 2 *eq*) and [2-(2-aminophenyl)phenyl]-chloro-palladium;bis(1-adamantyl)-butyl-phosphane (70.41 mg, 105.30 umol, 0.1 *eq*) in EtOH (2 mL) was de-gassed and then heated to 80 °C for 12 hr under N_2_. The reaction mixture was poured into H_2_O (5 mL). The mixture was extracted with ethyl acetate (10 mL*3). The organic phase was washed with brine (10 mL), dried over anhydrous Na2SO_4_, concentrated in vacuum to give a residue, which was purified by prep-HPLC (column: Welch Xtimate C18 100*25mm*3um;mobile phase: [water(0.05%HCl)-ACN];B%: 5%-25%,8min). Compound 6-ethyl-5-[3-(trifluoromethyl)phenyl]pyrimidine-2,4-diamine **WCDD115** (60 mg, 212.57 umol, 20.19% yield, 100% purity) was obtained as a white solid. MS: *m/z* 283.1 (M+H)^+^. ^1^H NMR (400 MHz): DMSO δ 7.807-7.827 (dd, J = 8 Hz, 1H), 7.726-7.765 (dt, J = 15.6 Hz, 1H), 7.646 (s, 1H), 7.578-7.597 (dd, J = 7.6 Hz, 1H), 2.357-2.300 (q, J = 22.8 Hz, 2H), 1.119-1.157 (t, J = 15.2 Hz, 3H). ^13^C NMR (400 MHz): MeOD_4_ δ 167.37, 162.71, 162.09, 136.33, 134.50, 131.3-130.97 (q, CCF_3_), 129.77, 127.25, 125.50-122.8 (q, CCF_3_), 124.36, 106.30, 27.31, 12.29.

6-ethyl-5-(3-methylsulfonylphenyl)pyrimidine-2,4-diamine (**WCDD118**)

To a solution of 4,4,5,5-tetramethyl-2-(3-methylsulfonylphenyl)-1,3,2-dioxaborolane **WCDD118_1** (200 mg, 708.81 umol, 1 *eq*) and 6-ethyl-5-iodo-pyrimidine-2,4-diamine **WCDD114_2** (196.53 mg, 744.25 umol, 1.05 *eq*) in THF (10 mL) was added ditert-butyl(cyclopentyl)phosphane;dichloropalladium;iron (46.20 mg, 70.88 umol, 0.1 *eq*) and K3PO4 (300.91 mg, 1.42 mmol, 2 *eq*) .The mixture was stirred at 80 °C for 12 hr . The reaction mixture was diluted with H_2_O (20 mL) and extracted with EtOAc (20 mL * 3). The combined organic layers were washed with EtOAc (20mL * 3), dried over Na_2_SO_4_, filtered and concentrated under reduced pressure to give a residue, which was purified by prep-HPLC twice. (column: Welch Xtimate C18 100*25mm*3um;mobile phase: [water(0.05%HCl)-ACN];B%: 1%-15%,8min) and (column: Waters Xbridge Prep OBD C18 150*40mm*10um;mobile phase: [water(10mM NH4HCO3)-ACN];B%: 5%-35%,8min.). Compound 6-ethyl-5-(3-methylsulfonylphenyl)pyrimidine-2,4-diamine **WCDD118** (32 mg, 109.05 umol, 15.39% yield, 99.63% purity) was obtained as a white solid. MS: *m/z* 293.1 (M+H)^+^. ^1^H NMR (400 MHz): DMSO δ 7.870-7.889 (dd, J = 7.6 Hz, 1H), 7.766-7.712 (dt, J = 8.3 Hz, 1H), 7.522-7.541 (dd, J = 7.6 Hz, 1H), 5.944 (s, 2H), 5.693 (br s, 2H), 3.236 (s, 3H), 2.056-2.122 (q, J = 26.4 Hz, 2H), 0.939-977 (t, J = 15.2 Hz, 3H),

6-ethyl-5-indan-5-yl-pyrimidine-2,4-diamine (**WCDD119**)

To a solution of indan-5-ylboronic acid **WCDD119_1** (200 mg, 1.23 mmol, 1 *eq*) and 6-ethyl-5-iodo-pyrimidine-2,4-diamine **WCDD114_2** (489.03 mg, 1.85 mmol, 1.5 *eq*) in dioxane (8 mL) and Water (2 mL) was added K2CO3 (341.26 mg, 2.47 mmol, 2 *eq*) and Pd(dppf)Cl2 (90.34 mg, 123.46 umol, 0.1 *eq*) .The mixture was stirred at 80 °C for 8 hr. The reaction mixture was diluted with H_2_O (20 mL ) and extracted with EtOAc (20 mL * 3). The combined organic layers were washed with EtOAc (20 mL * 3), dried over Na_2_SO_4_, filtered and concentrated under reduced pressure to give a residue, which was purified by prep-HPLC. (column: Welch Xtimate C18 100*25mm*3um;mobile phase: [water(0.05%HCl)-ACN];B%: 10%-40%,8min.). Compound 6-ethyl-5-indan-5-yl-pyrimidine-2,4-diamine **WCDD119** (59 mg, 201.23 umol, 16.30% yield, 99.18% purity, HCl) was obtained as a white solid. MS: *m/z* 255.1 (M+H)^+^. ^1^H NMR (400 MHz): DMSO δ 12.798 (s, 1H), 8.10 (s, 1H), 7.6 (br s, 2H), 7.317-7.366 (dd, 1H), 6.977 (s, 1H), 6.958-6.960 (s, 1H), 6.6 (s, 1H), 2.863-2.941 (m, 4H), 2.179-2.941 (m, 4H), 2.863-2.941 (m, 4H), 2.863-2.941 (m, 4H),

5-(2,5-dichlorophenyl)-6-ethyl-pyrimidine-2,4-diamine (**WCDD120**)

To a solution of 6-ethyl-5-iodo-pyrimidine-2,4-diamine (253.71 mg, 960.77 umol, 1 *eq*) in THF (1 mL) and H2O (0.25 mL) was added (2,5-dichlorophenyl)boronic acid (220 mg, 1.15 mmol, 1.2 *eq*) , [2-(2-aminophenyl)phenyl]-chloro-palladium;bis(1-adamantyl)-butyl-phosphane (64.24 mg, 96.08 umol, 0.1 *eq*) , K3PO4 (815.75 mg, 3.84 mmol, 4 *eq*) at 25°C. The mixture was stirred at 80 °C for 8 hr. The mixture was filtered and poured into water (50 mL), and extracted with ethyl acetate (50 mL*3). The organic layer was washed with brine (50 mL), and dried by MgSO_4_. The filtrate was concentrated to give the crude product, which was purified by prep-HPLC (column: Phenomenex luna C18 80*40mm*3 um;mobile phase: [water(0.04%HCl)-ACN];B%: 10%-38%,7min) to give 5-(2,5-dichlorophenyl)-6-ethyl-pyrimidine-2,4-diamine **WCDD120** (33 mg, 116.54 umol, 12.13% yield, 100% purity) as yellow solid. MS: *m/z* 283.0 (M+H)^+^. ^1^H NMR (400 MHz): DMSO δ 12.689 (s, 1H), 8.17 (br s, J = 8.3 Hz, 1H), 7.582-7.671 (dd, 2H), 7.539-7.589 (dt, 3H), 7.177 (br s, 1H), 2.072-2.263 (m, 2H), 0.989-1.073 (t, 3H)

5-(3-chloro-2-fluoro-phenyl)-6-ethyl-pyrimidine-2,4-diamine (**WCDD121**)

To a solution of 2-(3-chloro-2-fluoro-phenyl)-4,4,5,5-tetramethyl-1,3,2-dioxaborolane **WCDD121_1** (200 mg, 779.70 umol, 1 *eq*) in EtOH (5 mL) was added 6-ethyl-5-iodo-pyrimidine-2,4-diamine **WCDD114_2** (247.07 mg, 935.64 umol, 1.2 *eq*) and [2-(2-aminophenyl)phenyl]-chloro-palladium;bis(1-adamantyl)-butyl-phosphane (52.13 mg, 77.97 umol, 0.1 *eq*) and K3PO4 (331.01 mg, 1.56 mmol, 2 *eq*). The mixture was stirred at 80 °C for 12 hr. The reaction mixture was quenched by addition solvent NH_4_Cl 30 mL at 20 °C, and then extracted with e[thyl acetate](https://api.box.com/javascript:void(0);) 60 mL (20 mL * 3). The combined organic layers were washed with brine 40 mL (20 mL * 2), dried over by MgSO_4_, filtered and concentrated under reduced pressure to give a residue, which was purified by prep-HPLC (column: Phenomenex luna C18 80*40mm*3 um;mobile phase: [water(0.04%HCl)-ACN];B%: 10%-38%,7min). Compound 5-(3-chloro-2-fluoro-phenyl)-6-ethyl-pyrimidine-2,4-diamine **WCDD121** (27 mg, 88.76 umol, 11.38% yield, 99.66% purity, HCl) was obtained as a white solid. MS: *m/z* 267.1 (M+H)^+^. ^1^H NMR (400 MHz): DMSO δ 7.629-7.671 (td, 1H), 7.297-7.353 (m, 2H), 2.302-2.385 (m, 2H), 1.120-1.158 (t, 3H)

6-ethyl-5-(3-pyrrolidin-1-ylphenyl)pyrimidine-2,4-diamine (**WCDD122**)

To a solution of 1-[3-(4,4,5,5-tetramethyl-1,3,2-dioxaborolan-2-yl)phenyl]pyrrolidine **WCDD122_1** (200 mg, 732.13 umol, 1 *eq*) in EtOH (5 mL) was added 6-ethyl-5-iodo-pyrimidine-2,4-diamine **WCDD114_2** (386.66 mg, 1.46 mmol, 2 *eq*) and [2-(2-aminophenyl)phenyl]-chloro-palladium;bis(1-adamantyl)-butyl-phosphane (48.95 mg, 73.21 umol, 0.1 *eq*) and K3PO4 (310.81 mg, 1.46 mmol, 2 *eq*) .The mixture was stirred at 80 °C for 12 hr. The reaction mixture was quenched by addition solvent NH_4_Cl 30 mL at 20 °C, and then extracted with e[thyl acetate](https://api.box.com/javascript:void(0);) 60 mL (20 mL * 3). The combined organic layers were washed with brine 40 mL (20 mL * 2), dried over by MgSO_4_, filtered and concentrated under reduced pressure to give a residue, which was purified by prep-HPLC (column: Phenomenex luna C18 80*40mm*3 um;mobile phase: [water(0.04%HCl)-ACN];B%: 10%-38%,7min). Compound 6-ethyl-5-(3-pyrrolidin-1-ylphenyl)pyrimidine-2,4-diamine **WCDD122** (27 mg, 81.88 umol, 11.18% yield, 96.99% purity, HCl) was obtained as a yellow oil. MS: *m/z* 284.2 (M+H)^+^. ^1^H NMR (400 MHz): DMSO δ 7.728-7.740 (dd, 2H), 7.614 (s, 1H), 7.431-7.449 (m, 1H), 3.841 (m, 4H), 2.310-2.386 (m, 6H), 1.135-1.200 (t, 3H).

6-ethyl-5-(3-morpholinophenyl)pyrimidine-2,4-diamine (**WCDD123**)

To a solution of 4-[3-(4,4,5,5-tetramethyl-1,3,2-dioxaborolan-2-yl)phenyl]morpholine **WCDD123_1** (100 mg, 345.81 umol, 1 *eq*) , 6-ethyl-5-iodo-pyrimidine-2,4-diamine **WCDD114_2** (109.58 mg, 414.97 umol, 1.2 *eq*) , K3PO4 (146.81 mg, 691.62 umol, 2 *eq*) and [2-(2-aminophenyl)phenyl]-chloro-palladium;bis(1-adamantyl)-butyl-phosphane (23.12 mg, 34.58 umol, 0.1 *eq*) in EtOH (10 mL) at 25°C. The mixture was stirred at 80 °C for 12 hr. The mixture was filtered, and the filtrate was concentrated to give the crude product, which was purified by pre-HPLC (column: Welch Xtimate C18 100*25mm*3um;mobile phase: [water(0.05%HCl)-ACN];B%: 1%-20%,8min)(column: Phenomenex luna C18 80*40mm*3 um;mobile phase: [water(0.04%HCl)-ACN];B%: 12%-26%,6.5min) to give 6-ethyl-5-(3-morpholinophenyl)pyrimidine-2,4-diamine (14 mg, 46.76 umol, 13.52% yield) as a yellow solid. MS: *m/z* 300.2 (M+H)^+^. ^1^H NMR (400 MHz): DMSO δ 7.703-7.782 (td, 2H), 7.643 (s, 1H), 7.385-7.404 (dd, 1H), 4.103-4.126 (t, 4H), 3.675-3.683 (d, 4H), 2.337-2.394 (q, 2H), 1.137-1.174 (t, 3H).

5-(3-chlorophenyl)-6-ethyl-pyrimidin-4-amine (**WCDD125**)

A solution of 4-chloro-6-ethyl-pyrimidine **WCDD125_1** (0.5 g, 3.51 mmol, 1 *eq*) in NH3.H2O (20 mL) was stirred at 100 °C for 12 hr. The mixture was concentrated to give the product 6-ethylpyrimidin-4-amine **WCDD125_2** (0.4 g, 3.25 mmol, 92.62% yield) as white solid. ^1^H NMR (400 MHz): DMSO-d6 δ 8.32 (s, 1H), 7.09 (s, 2H), 6.31 (s, 1H), 2.46 (dd, 2H), 1.13 (t, 3H).

To a solution of 6-ethylpyrimidin-4-amine **WCDD125_2** (370 mg, 3.00 mmol, 1 *eq*) in DMF (1.5 mL) was added NBS (641.65 mg, 3.61 mmol, 1.2 *eq*) at 25 °C for 2 hr. The mixture was poured into saturated NH_4_Cl solution (100 mL) and extracted with EtOAc (50 mL*3). The organic layer was washed with brine (100 mL*3), dried by MgSO_4_, and filtered. The filtrate was concentrated to give the product 5-bromo-6-ethyl-pyrimidin-4-amine **WCDD125_3** (400 mg, 1.58 mmol, 52.72% yield, 80% purity) as white solid. ^1^H NMR (400 MHz): DMSO δ 8.26 (s, 1H), 8.06 (d, J = 8.3 Hz, 1H), 6.98 (s, 2H), 2.68 (m, 2H), 1.13 (t, 3H).

5-bromo-6-ethyl-pyrimidin-4-amine **WCDD125_3** (0.165 g, 816.62 umol, 1 *eq*) , (3-chlorophenyl)boronic acid **WCDD125_4** (153.24 mg, 979.95 umol, 1.2 *eq*) , Pd(dppf)Cl2.CH2Cl2 (100.03 mg, 122.49 umol, 0.15 *eq*) and Cs2CO3 (532.14 mg, 1.63 mmol, 2 *eq*) were taken up into a microwave tube in H2O (1.125 mL) , dioxane (3 mL)The sealed tube was heated at 100 °C for 12 hr under microwave. After cooling to 0°C, ethyl acetate (30 mL) and H_2_O (20 mL) were added. The aqueous layer was extracted with ethyl acetate (30 mL*3). The combined organic layers were washed with brine (30 mL), dried over Na_2_SO_4_, concentrated in vacuo to give the crude product, which was purified by prep-HPLC (column: Kromasil C18 (250*50mm*10 um);mobile phase: [water(10mM NH4HCO3)-ACN];B%: 20%-50%,10min) to give 5-(3-chlorophenyl)-6-ethyl-pyrimidin-4-amine **WCDD125** (102 mg, 425.90 umol, 52.15% yield, 97.58% purity) as a white solid. MS: *m/z* 232.1 (M-H)^+^. ^1^H NMR (400 MHz): DMSO δ 8.37 (s, 1H), 7.49 (m, 2H), 7.30 (s, 1H), 7.18 (d, 1H), 6.18 (s, 2H), 2.21 (q, 2H), 1.00 (t, 3H).

5-(3-chlorophenyl)-6-[2-(2-methoxyethoxy)ethyl]pyrimidine-2,4-diamine (**WCDD126**)

To a solution of 3-(2-methoxyethoxy)propanoic acid **WCDD126_1** (0.8 g, 5.40 mmol, 1 *eq*) in DCM (24 mL) was added SOCl2 (963.60 mg, 8.10 mmol, 587.56 uL, 1.5 *eq*) and DMF (19.73 mg, 269.98 umol, 20.77 uL, 0.05 *eq*) at 0°C. The mixture was stirred at 0~20 °C for 3 hr. The mixture was concentrated to give the crude product 3-(2-methoxyethoxy)propanoyl chloride **WCDD126_2** (0.8 g, 4.56 mmol, 84.48% yield, 95% purity) as a light yellow oil, which was used directly for the next step.

To a solution of 2-(3-chlorophenyl)acetonitrile **WCDD126_5** (727.93 mg, 4.80 mmol, 568.69 uL, 1 *eq*) in dry THF (8 mL) was added a solution of LDA (2 M, 2.40 mL, 1 *eq*) in dry THF (8 mL) dropwise at -70 °C . The mixture was stirred at -70 °C for 1 hr. A solution of 3-(2-methoxyethoxy)propanoyl chloride **WCDD126_2** (0.8 g, 4.80 mmol, 1 *eq*) in dry THF (2 mL) was added dropwise at -70 °C and stirred for 1 hr. The reaction mixture was quenched with cold saturated aqueous NH_4_Cl (100 mL), extracted with EtOAc (50 mL x 3). The organic layer was washed with brine (40 mL), dried over Na_2_SO_4_, filtered and concentrated to dryness under reduced pressure. The residue was purified by flash silica gel chromatography (ISCO®; 12 g SepaFlash® Silica Flash Column, Eluent of 0~55% Ethyl acetate/Petroleum ethergradient @ 40 mL/min). 2-(3-chlorophenyl)-5-(2-methoxyethoxy)-3-oxo-pentanenitrile **WCDD126_3** (0.38 g, 809.27 umol, 16.85% yield, 60% purity) was obtained as light yellow oil. ^1^H NMR (400 MHz): CDCl­_3_ δ 9.29 (s, 1H), 7.81 (t, 1H), 7.58 (dd, 1H), 7.39 (m, 1H), 7.28 (m, 1H), 7.21 (d, 1H), 4.25 (dd, 1H), 3.73-3.79 (m, 4H), 3.59 (m, 6H), 3.44 (m, 3H), 3.39 (d, 5H), 3.04 (m, 2H), 2.63 (q, 2H), 1.36 (d, 3H), 1.18 (d, 3H).

A solution of 2-(3-chlorophenyl)-5-(2-methoxyethoxy)-3-oxo-pentanenitrile **WCDD126_3** (380 mg, 809.27 umol, 60% purity, 1 *eq*) in 1,1,1-trimethoxyethane (1.21 g, 10.03 mmol, 1.26 mL, 12.4 *eq*) was heated to 100 °C and stirred for 12 hr. After concentration, the residue was purified by flash silica gel chromatography (ISCO®; 12 g SepaFlash® Silica Flash Column, Eluent of 0~30% Ethyl acetate/Petroleum ethergradient @ 20 mL/min) to give (Z)-2-(3-chlorophenyl)-3-methoxy-5-(2-methoxyethoxy)pent-2-enenitrile **WCDD126_4** (0.15 g, 507.17 umol, 62.67% yield) as light yellow oil. MS: *m/z* 296.2 (M+H)^+^.

To a solution of (Z)-2-(3-chlorophenyl)-3-methoxy-5-(2-methoxyethoxy)pent-2-enenitrile **WCDD126_4** (0.15 g, 507.17 umol, 1 *eq*) and guanidine;hydrochloride **WCDD126_6** (96.90 mg, 1.01 mmol, 81.43 uL, 2 *eq*) in dry DMSO (1.8 mL) was added NaHCO3 (93.73 mg, 1.12 mmol, 43.39 uL, 2.2 *eq*) . The mixture was heated to 80 °C and stirred for 12 hr. The reaction mixture was cooled to 20 °C, filtered and the cake was washed with EtOAc (3 mL x 3). The filtrate was concentrated under reduced pressure. The residue was purified by prep-HPLC (neutral condition, column: Phenomenex Gemini-NX 80*40mm*3um;mobile phase: [water(10mM NH4HCO3)-ACN];B%: 20%) to give 5-(3-chlorophenyl)-6-[2-(2-methoxyethoxy)ethyl]pyrimidine-2,4-diamine **WCDD126** (21 mg, 63.95 umol, 12.61% yield, 98.30% purity) as off-white solid. MS: *m/z* 323.1 (M-H)^+^. ^1^H NMR (400 MHz): CDCl_3_ δ 7.35 (dd, 2H), 7.29 (d, 1H), 7.15 (d, 1H), 4.70 (s, 1H), 4.45 (s, 1H), 3.71 (t, 2H), 3.50 (m, 4H), 3.35 (s, 3H), 2.59 (t, 2H).

5-(3-chlorophenyl)-6-(cyclobutylmethyl)pyrimidine-2,4-diamine (**WCDD127**)

2-cyclobutylacetic acid **WCDD127_1** (1.2 g, 10.51 mmol, 1 *eq*) was dissolved in DCM (5 mL) containing 2 drops DMF (76.84 mg, 1.05 mmol, 80.89 uL, 0.1 *eq*), and oxalyl dichloride (1.60 g, 12.62 mmol, 1.10 mL, 1.2 *eq*) was added drop wise over 3 min at 25°C. The reaction mixture was stirred at 25°C for 3 hr. Then the solvent was removed to give a residue. To a solution of LDA (1 M, 14.72 mL, 1.4 *eq*) in DCM (5 mL) was added dropwise 2-(3-chlorophenyl)acetonitrile **WCDD126_5** (1.91 g, 12.62 mmol, 1.49 mL, 1.2 *eq*) at -70°C over 10 mins. After addition, the mixture was stirred at this temperature for 30min, and then the former residue in THF (10 mL) was added dropwise at -70°C. The resulting mixture was stirred at 25 °C for 3 hr. The reaction mixture was poured into NH4Cl 20 mL at 0°C, and then extracted with ethyl acetate (20 mL * 3). The combined organic layers were washed with brine (20 mL * 2), dried over MgSO4, filtered and concentrated under reduced pressure to give a residue, which was purified by flash silica gel chromatography (ISCO®; 10 g SepaFlash® Silica Flash Column, Eluent of 0~30% Ethyl acetate/Petroleum ethergradient @ 100 mL/min) to give 2-(3-chlorophenyl)-4-cyclobutyl-3-oxo-butanenitrile **WCDD127_3** (1.4 g, 5.65 mmol, 53.76% yield) as a brown oil. ^1^H NMR (400 MHz): DMSO-d6 δ 11.89 (s, 1H), 7.54 (s, 1H), 7.52 (d, 1H), 7.39 (t, 1H), 7.26 (d, 1H), 2.69 (s, 3H), 2.06 (d, 2H), 1.84 (m, 4H).

A solution of 2-(3-chlorophenyl)-4-cyclobutyl-3-oxo-butanenitrile **WCDD127_3** (1.1 g, 4.44 mmol, 1 *eq*) in 1,1,1-trimethoxyethane (6.57 g, 54.70 mmol, 6.88 mL, 12.32 *eq*) was degassed and purged with N_2_ for 3 times, and then the mixture was stirred at 107 °C for 6 hr under N_2_ atmosphere. The reaction mixture was concentrated under reduced pressure to remove solvent. The residue was diluted with H_2_O 50 mL and extracted with ethyl acetate (50 mL * 3). The combined organic layers were washed with brine (100 mL * 2), dried over MgSO_4_, filtered and concentrated under reduced pressure to give crude product, which was purified by flash silica gel chromatography (ISCO®; 10 g SepaFlash® Silica Flash Column, Eluent of 0~30% Ethyl acetate/Petroleum ethergradient @ 100 mL/min) to give (Z)-2-(3-chlorophenyl)-4-cyclobutyl-3-methoxy-but-2-enenitrile **WCDD127_4** (0.6 g, 2.29 mmol, 51.62% yield) as an orange solid. ^1^H NMR (400 MHz, DMSO-d_6_) δ 7.56 (t, *J*=1.83 Hz, 1H), 7.44-7.51 (m, 1H), 7.37-7.44 (m, 1H), 7.31-7.36 (m, 1H), 3.82-3.94 (m, 3H), 2.90 (d, *J*=7.34 Hz, 2H), 2.59-2.76 (m, 1H), 2.04-2.18 (m, 2H), 1.78-1.99 (m, 4H)

To a solution of (Z)-2-(3-chlorophenyl)-4-cyclobutyl-3-methoxy-but-2-enenitrile **WCDD127_4** (0.4 g, 1.53 mmol, 1 *eq*) in DMSO (5 mL) was added NaHCO3 (282.44 mg, 3.36 mmol, 130.76 uL, 2.2 *eq*) and guanidine;hydrochloride **WCDD126_6** (291.98 mg, 3.06 mmol, 245.36 uL, 2 *eq*). The mixture was stirred at 80 °C for 5 hr. The reaction mixture was partitioned between ethyl acetate 30 mL and water 10 mL. The organic phase was separated, washed with water (20 mL * 3), dried over MgSO4, filtered and concentrated under reduced pressure to give a residue, which was purified by prep-HPLC (neutral condition column: Kromasil C18 (250*50mm*10 um);mobile phase: [water(10mM NH4HCO3)-ACN];B%: 30%-70%,10min) to give 5-(3-chlorophenyl)-6-(cyclobutylmethyl)pyrimidine-2,4-diamine **WCDD127** (0.066 g, 227.84 umol, 14.91% yield, 99.69% purity) as a white solid. MS: *m/z* 289.2 (M+H)^+^. ^1^H NMR (400 MHz): DMSO-d6 δ 7.45 (dd, 1H), 7.41 (d, 1H), 7.18 (s, 1H), 7.10 (dd, 1H), 5.84 (s, 2H), 5.55 (s, 2H), 2.51 (td, 1H), 2.20 (d, 2H), 1.86 (m, 2H), 1.67 (m, 2H), 1.43 (m, 2H).

5-(3-chlorophenyl)-6-pent-4-ynyl-pyrimidine-2,4-diamine (**WCDD128**)

To a solution of hex-5-ynoic acid **WCDD128_1** (1 g, 8.92 mmol, 970.87 uL, 1 *eq*) in dry THF (20 mL) were added (COCl)2 (2.26 g, 17.84 mmol, 1.56 mL, 2 *eq*) and DMF (13.04 mg, 178.37 umol, 13.72 uL, 0.02 *eq*) . The solution was stirred at 25 °C for 3 hr. The reaction mixture was concentrated to dryness under reduced pressure to give hex-5-ynoyl chloride **WCDD128_2** (1.1 g, crude) as yellow oil, which was used for the next step directly.

To a solution of LDA (2 M, 2.97 mL, 1 *eq*) in dry THF (9 mL) was added a solution of 2-(3-chlorophenyl)acetonitrile **WCDD126_5** (900 mg, 5.94 mmol, 703.13 uL, 1 *eq*) in dry THF (9 mL) at -70 °C under N_2_. The mixture was stirred at -70 °C for 1 hr. A solution of hex-5-ynoyl chloride **WCDD128_2** (775.20 mg, 5.94 mmol, 1 *eq*) in dry THF (2.25 mL) was added. The mixture was stirred for another 1 hr and then poured into saturated aqueous NH_4_Cl (100 mL), extracted with EtOAc (50 mL x 3). The organic layer was washed with brine (50 mL), dried over anhydrous Na_2_SO_4_, filtered and concentrated to dryness under reduced pressure. The residue was purified by flash silica gel chromatography (ISCO®; 12 g SepaFlash® Silica Flash Column, Eluent of 0~40% Ethyl acetate/Petroleum ethergradient @ 15 mL/min) to give 2-(3-chlorophenyl)-3-oxo-oct-7-ynenitrile **WCDD128_3** (0.3 g, 1.22 mmol, 20.57% yield) as light yellow solid. ^1^H NMR (400 MHz): CDCl_3_ δ 7.38-7.41 (m, 3H), 7.29-7.31 (d, 1H), 4.86 (s, 1H), 2.81 (m, 2H), 2.19 (td, 2H), 1.94 (s, 1H), 1.79 (m, 2H).

A solution of 2-(3-chlorophenyl)-3-oxo-oct-7-ynenitrile **WCDD128_3** (0.3 g, 1.22 mmol, 1 *eq*) in 1,1,1-trimethoxyethane (1.82 g, 15.14 mmol, 1.90 mL, 12.4 *eq*) was degassed and purged with N_2_ for seneral times. The mixture was heated to 100 °C and stirred for 12 hr. The reaction mixture was cooled to 25 °C, concentrated under reduced pressure. The residue was diluted with EtOAc (20 mL), washed with H_2_O (6 mL) and brine (10 mL), dried over Na_2_SO_4_, filtered and concentrated to dryness under reduced pressure. The residue was purified by prep-TLC (SiO2, petroleum ether: ethyl acetate = 3:1) to give (Z)-2-(3-chlorophenyl)-3-methoxy-oct-2-en-7-ynenitrile **WCDD128_4** (0.11 g, 381.16 umol, 31.22% yield, 90% purity) as yellow oil. ^1^H NMR (400 MHz): CDCl_3_ δ 7.66 (s, 1H), 7.47-7.65 (d, 1H), 7.24-7.31 (m, 2H), 3.91 (s, 3H), 2.89-2.93 (m, 2H), 2.38-2.40 (t, 2H), 2.06 (t, 2H), 1.90-1.94 (m, 2H).

To a mixture of (Z)-2-(3-chlorophenyl)-3-methoxy-oct-2-en-7-ynenitrile **WCDD128_4** (110 mg, 381.16 umol, 90% purity, 1 *eq*) and guanidine **WCDD126_6** (72.83 mg, 762.33 umol, 2 *eq*, HCl) in dry DMSO (1.3 mL) was added NaHCO3 (70.44 mg, 838.56 umol, 32.61 uL, 2.2 *eq*)The mixture was heated to 80 °C and stirred for 12 hr. The reaction mixture was cooled to 20 °C, diluted with EtOAc (10 mL), filtered. The cake was washed with EtOAc (3 x 3 mL) and the filtrate was concentrated to dryness under reduced pressure. The residue was purified by prep-HPLC (HCl, condition: column: Welch Xtimate C18 100*25mm*3um;mobile phase: [water(0.05%HCl)-ACN];B%: 15%-45%,8min) to give 5-(3-chlorophenyl)-6-pent-4-ynyl-pyrimidine-2,4-diamine **WCDD128** (53 mg, 183.60 umol, 48.17% yield, 99.34% purity) as off-white solid. MS: *m/z* 287.1 (M+H)^+^. ^1^H NMR (400 MHz): MeOD δ 7.51 (dd, 2H), 7.38 (s, 1H), 7.25 (m, 1H), 2.47 (td, 2H), 2.15 (m, 3H), 1.73 (m, 2H).

5-(4-chlorophenyl)-6-ethyl-N2,N4-dimethyl-pyrimidine-2,4-diamine (**WCDD133**)

To a solution of 5-(4-chlorophenyl)-6-ethyl-pyrimidine-2,4-diamine **WCDD133_1** (0.5 g, 2.01 mmol, 1 *eq*) in THF (20 mL) was added NaH (168.87 mg, 4.22 mmol, 60% purity, 2.1 *eq*) at 0°C for 30 min. And then MeI (627.77 mg, 4.42 mmol, 275.34 uL, 2.2 *eq*) was added into the mixture at 0°C. The mixture was stirred at 25 °C for 8 hr.

The reaction mixture was quenched by addition NH_4_Cl (40 mL) at 0°C, and then diluted with H_2_O (10 mL) and extracted with EtOAc (50 mL * 3). The combined organic layers were washed with EtOAc (30 mL * 3), dried over Na_2_SO_4_, filtered and concentrated under reduced pressure to give a residue, which was purified by prep-HPLC (column: Welch Xtimate C18 100*25mm*3um;mobile phase: [water(0.05%HCl)-ACN];B%: 10%-40%,8min.) to give 5-(4-chlorophenyl)-6-ethyl-N2,N4-dimethyl-pyrimidine-2,4-diamine **WCDD133** (33 mg, 100.31 umol, 4.99% yield, 95.21% purity, HCl) as a white solid, and 5-(4-chlorophenyl)-6-ethyl-N4-methyl-pyrimidine-2,4-diamine **WCDD133a** (15 mg, 49.14 umol, 2.44% yield, 98.01% purity, HCl) as a white solid.

WCDD133: MS: *m/z* 277.1 (M+H)^+^. ^1^H NMR (400 MHz): DMSO-d6 δ 12.56 (s, 1H), 7.56-7.58 (d, 3H), 7.28-7.30 (d, 3H), 2.95-3.00 (d, 2H), 2.85-2.90 (d, 3H), 2.25 (m, 2H), 1.05 (t, 3 H).

WCDD133a: MS: *m/z* 263.1 (M+H)^+^. ^1^H NMR (400 MHz): DMSO-d6 δ 12.58 (s, 1H), 7.56-7.58 (d, 3H), 7.22-7.30 (d, 3H), 2.77-2.79 (d, 3H), 2.20 (m, 2H), 1.04 (t, 3 H).
